# A rare case of endoscopic full-thickness resection of a laterally spreading tumor at the anastomotic site of appendectomy

**DOI:** 10.1055/a-2248-0579

**Published:** 2024-02-22

**Authors:** Yinong Zhu, Wei Liu, Bing Hu

**Affiliations:** 1 34753 Department of Gastroenterology and Hepatology, West China Hospital of Sichuan University, Chengdu, China

**Fig. 1:**
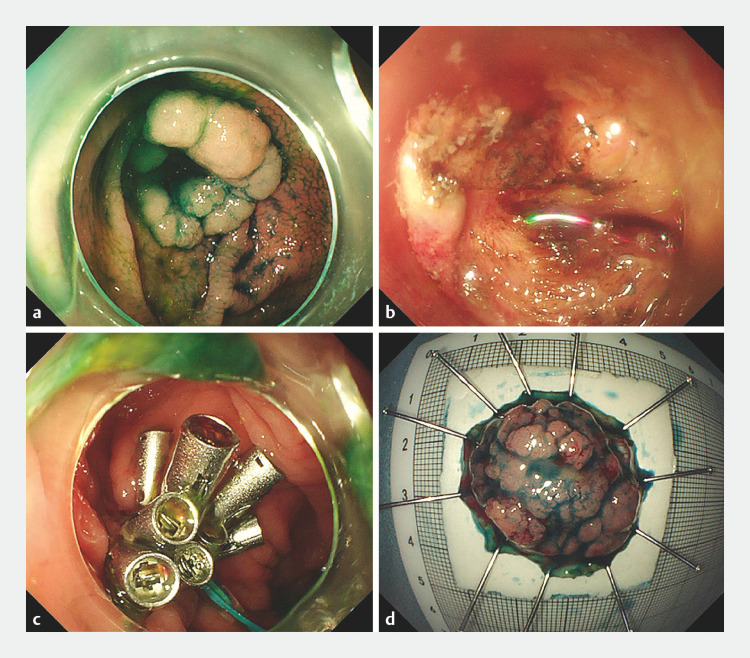
Successful en bloc resection of a laterally spreading tumor at the anastomotic site of appendectomy. **a** Colonoscopy revealed a laterally spreading tumor approximately 25  mm in diameter at the appendiceal orifice. **b** During the procedure, submucosal tissue adhesion was observed. **c** The postoperative defect was closed. **d** Full-thickness resection specimen.

A 67-year-old man who had undergone an appendectomy 20 years ago was admitted owing to the presence of a laterally spreading tumor (LST) at the anastomotic site of the previous surgery ( Fig. 1 **a** , Video 1 ). After a thorough preoperative examination and considering the inherent difficulty of performing endoscopic submucosal dissection (ESD) at the appendiceal orifice and the additional challenge posed by surgical anastomotic scar adhesions, endoscopic full-thickness resection (EFTR) was chosen as the treatment modality. During the procedure, a C-shaped incision revealed a severe postoperative tissue adhesion ( Fig. 1 **b** ), confirming the difficulty associated with ESD. Utilizing the initial C-shaped incision, partial dissection of the submucosal layer was performed, followed by a circumferential incision of the lesion mucosa using the DualKnife (Olympus, Tokyo, Japan). After that, an IT knife (Olympus) was used to complete the endoscopic full-thickness resection. The defect was closed using nylon cords (Micro-Tech, Nanjing, China) and 14 titanium clips ( Fig. 1 **c** , Fig. 1 **d** ). The patient was discharged 4 days after the treatment, and the pathology revealed a tubular adenoma.

A rare case of endoscopic full-thickness resection of a laterally spreading tumor at the anastomotic site of appendectomy.Video 1

It should be noted that performing ESD on the appendiceal orifice poses inherent challenges, and performing resection at the anastomotic site further complicates the procedure owing to postoperative tissue adhesion. Limited reports exist on ESD performed at the appendiceal orifice. Furthermore, to date, there have been no reports on endoscopic treatments conducted at the appendiceal orifice with postsurgical anastomosis. In this particular case, considering the lesionʼs location within the surgical scar, full-thickness resection was deemed the optimal approach to overcome difficulties associated with poor elevation and unclear cutting planes. The successful execution of this procedure provides valuable insights for managing similar cases in the future.

Endoscopy_UCTN_Code_TTT_1AQ_2AD

**Correction: A rare case of endoscopic full-thickness resection of a laterally spreading tumor at the anastomotic site of appendectomy**

**Yinong Zhu, Wei Liu, Bing Hu. A rare case of endoscopic full-thickness resection of a laterally spreading tumor at the anastomotic site of appendectomy.** Endoscopy 2024; 56: E199–E200, doi:10.1055/a-2248-0579

In the above-mentioned article the authorship has been corrected. Correct is that Yinong Zhu and Wei Liu contribute equally as first authors. This was corrected in the online version on March 22, 2024.

