# Correction: A rare case of endoscopic full-thickness resection of a laterally spreading tumor at the anastomotic site of appendectomy

**DOI:** 10.1055/a-2292-4127

**Published:** 2024-03-26

**Authors:** Yinong Zhu, Wei Liu, Bing Hu

**Affiliations:** 1 34753 Department of Gastroenterology and Hepatology, West China Hospital of Sichuan University, Chengdu, China



In the above-mentioned article the authorship has been corrected. Correct is that Yinong Zhu and Wei Liu contribute equally as first authors. This was corrected in the online version on March 22, 2024.

